# Method Development and Validation for Pharmacokinetic and Tissue Distributions of Ellagic Acid Using Ultrahigh Performance Liquid Chromatography-Tandem Mass Spectrometry (UPLC-MS/MS)

**DOI:** 10.3390/molecules191118923

**Published:** 2014-11-18

**Authors:** Linlin Yan, Peipei Yin, Chao Ma, Yujun Liu

**Affiliations:** 1 National Engineering Laboratory for Tree Breeding, College of Biological Sciences and Biotechnology, Beijing Forestry University, Beijing 100083, China; E-Mails: yanlinlin155@163.com (L.Y.); happy62889@126.com (P.Y.); 2 Beijing Key Laboratory of Forest Food Processing and Safety, College of Biological Sciences and Biotechnology, Beijing Forestry University, Beijing 100083, China

**Keywords:** ellagic acid, solid phase extraction, pharmacokinetics, tissue distribution, UPLC-MS/MS

## Abstract

Ellagic acid is a dietary polyphenol found in numerous fruits and vegetables, possessing several health benefits such as antioxidant, anticancer and anti-atherosclerotic biological properties. The purpose of this study was to explore the pharmacokinetics and tissue distribution of ellagic acid in rats. A simple, rapid, sensitive and specific liquid chromatography–tandem mass spectrometry method to determine the ellagic acid in plasma and tissue samples was developed and validated. The separation was achieved using reversed-phase ultra-performance liquid chromatography (UPLC), and the mass spectrometric detection was achieved using heated electrospray ionization (negative mode) and multiple ion monitoring (*m*/*z* 301/229). A sample cleanup with a solid phase extraction (SPE) step prior to the UPLC-MS/MS analysis was also developed. The SPE and UPLC-MS/MS method established here was successfully applied to reveal the pharmacokinetic profiles and tissue distribution of ellagic acid. After oral administration dosing at 50 mg/kg, plasma levels of ellagic acid peaked at about 0.5 h, with *C*_max_ value of 93.6 ng/mL, and the results showed that the ellagic acid was poorly absorbed after oral administration. The pharmacokinetic profile of ellagic acid fitted to a two-compartment model with *t*_1/2α_ 0.25 h and *t*_1/2β_ 6.86 h, respectively. Following oral administration, ellagic acid was detected in all examined tissues including kidney, liver, heart, lung and brain *et al.*, and the highest levels were found in kidney and liver.

## 1. Introduction

Ellagic acid (EA) and hydrolysable ellagitannins (ETs) are dietary polyphenols found in numerous fruits, vegetables and nuts such as pomegranate, blackberry, raspberry, chestnut, and walnut [1,2]. In the gut, ETs are easily hydrolyzed to release EA units through the *in vivo* action of physiological pH and/or the enzymatic activity of gut microflora [3]. EA is known to possess many health benefits such as antioxidant, anticancer, antiatherosclerotic and other biological properties [1,2,3]. Therefore, elucidating the pharmacokinetic and tissue distribution profiles of EA is important, which can increase our understanding of the health beneficial mechanisms and provide more information for the scientific consumption of EA or EA/ETs-rich foods. The first study on the metabolism of EA was performed in rats after oral administration, and the concentrations of EA and its metabolites in urine and feces were reported [4]. Afterward, the tissue distribution of EA in mice after oral administration was studied [5]. In the following decades, many other pharmacokinetic studies have been performed following consumption of pomegranate leaf extract in rats [6], pomegranate juice in humans [7], and frozen dried black raspberries in healthy volunteers [8]. Based on the above studies, the absorption of EA in animals is very poor and usually undergoes extensive metabolism by the gut microflora to produce urolithins [9]. Furthermore, previous studies regarding the concentration of EA in blood and tissues have been controversial. For example, no EA was recovered from the blood or tissues of mice fed a diet containing 1.0% EA for 1 week [10], while Boukharta *et al.* [5] found that the levels of EA in blood was about 1 μM and localized preferentially in lung tissues after oral administration. In addition, Seeram *et al.* [7] reported that the maximum concentration of EA in human plasma was about 0.1 μM after consumption of pomegranate juice. These conflicts may be attributable to the lack of a sensitive method for the determination of EA, as well as its poor absorption and extensive metabolic transformation and degradation prior to absorption. Therefore, a sensitive and reproducible method for detecting EA in tissues or plasma is required.

Many analytical methods have been developed for this purpose, but most of these approaches were optimized for EA quantification in foods or extracts based on high-performance liquid chromatography (HPLC) with ultraviolet (UV) detection systems [11] or liquid chromatography-mass spectrometry (LC-MS) systems [12]. The development of suitable protocols for deproteinization, extraction, and analysis of EA in plasma or tissues, however, is challenging since EA is commonly non-covalently bound to cellular DNA/proteins [13] and is poorly absorbed. Although several methods have been developed based on organic solvent deproteinization, extraction protocols, and HPLC-UV analysis systems for pharmacokinetic and bio-distribution studies [5,6,7], they are typically complicated, time-consuming, or have limited sensitivity. Furthermore, a large number of samples are generally analyzed in pharmacokinetic and tissue distribution studies. Therefore, a rapid, simple, and high-throughput sample preparation protocol and a rapid, selective, and sensitive analytical method are required. In the present study, a solid phase extraction (SPE) sample preparation protocol together with an ultrahigh performance liquid chromatography coupled with tandem mass spectrometry (UPLC-MS/MS) method was developed to quantify EA in plasma and tissues. In addition, the validation parameters including linearity, selectivity, repeatability, reproducibility, recovery, decision limit, and detection capability of the method were determined.

## 2. Results and Discussion

### 2.1. Optimization of Sample Preparation

To develop a protocol for sample pre-preparation with high efficiency and improved recovery, different methods were compared. Firstly, the conventional organic solvent deproteinization and extraction method was used as follows: 400 μL of methanol or acetonitrile was added to 100 μL of blank plasma spiked with 1 μg of EA and vortex mixed for 5 min. The mixture was centrifuged at 4000 rpm for 10 min to obtain the supernatant. However, HPLC-DAD analysis revealed that the recoveries for methanol and acetonitrile treatment of EA in plasma were only 17.8% and 13.1%, respectively, therefore, conventional organic solvent deproteinization and extraction methods are evidently not suitable for EA detection in plasma. A SPE cartridge was then used for the plasma treatment. Firstly, 100 μL of blank plasma spiked with 1 μg EA was loaded on a preconditioned SPE cartridge, which was then eluted successively with 2 mL of water and 2 mL of methanol. However, almost no EA (with recovery of about 3.0%) was found in methanol eluate, which indicated that the majority of EA was absorbed in the cartridge after binding to proteins in plasma. Trichloroacetic acid (TCA), H_3_PO_4_, or NaOH was then added in the plasma to improve the recovery, as follows: 5 μL 20% TCA, 4 M NaOH, or 50% H_3_PO_4_ was added to 100 μL of blank plasma spiked with 1 μg EA, and after vortexing, the mixture was load on the SPE cartridges and eluted with 2 mL of water and 2 mL of methanol, successively. The recoveries of EA for TCA, NaOH, and H_3_PO_4_ treatment were 21.0%, 5.6%, and 50.9%, respectively. Finally, KH_2_PO_4_ was added in the H_3_PO_4_ treatment system, as follows: 5 μL of 50% H_3_PO_4_ and 35 μL of KH_2_PO_4_ were added successively to 100 μL of blank plasma spiked with 1 μg EA, and after vortexing, the mixture was loaded onto SPE and eluted as described above. The recovery of EA in the methanol eluate was 89.6%, which indicated that the addition of H_3_PO_4_ and KH_2_PO_4_ significantly reduced the non-covalent binding of EA with cellular DNA or proteins [13].

To further optimize the elution procedure for loaded SPE cartridges, a gradient elution program was designed as follows: 5 μL of 50% H_3_PO_4_ and 35 μL of KH_2_PO_4_ were added to 100 μL of blank plasma spiked with 0.5 μg EA, and after vortexing, the mixture was loaded onto a preconditioned SPE cartridge. Subsequently, the cartridge was eluted with 3 mL of deionized water, 3 mL of 40% (v/v) methanol in 0.1% formic acid aqueous solution, 0.5 mL of 90% (v/v) methanol in 0.1% formic acid aqueous solution, and 1 mL of methanol, successively. The mean recovery of EA in 0.5 mL of 90% methanol in 0.1% formic acid aqueous solution was 98.5% with RSD 0.84%, which indicated that the formic acid significantly improved the recovery of EA from cartridge. Therefore, the elution procedure was optimized as follows: after sample loading, the cartridge was washed with 3 mL of deionized water, 3 mL of washing solution (40% methanol in 0.1% formic acid aqueous solution), and 0.5 mL of elution solution (90% methanol in 0.1% formic acid aqueous solution).

### 2.2. Optimization of Chromatographic and MS/MS Conditions

**Figure 1:**
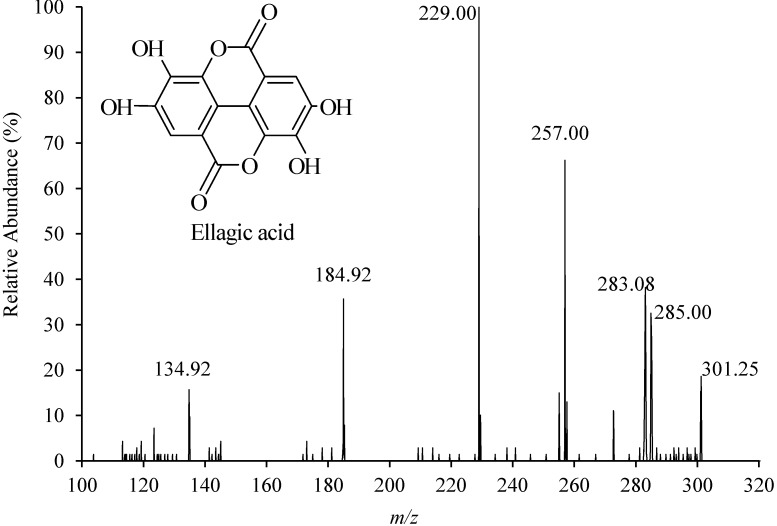
Negative electrospray ionization mass spectra and molecular structure of ellagic acid.

To achieve the maximum sensitivity, the MS parameters including ionization mode (positive, negative), the ion spray voltage (IS), source temperature (TEM), declustering potential (DP), collision energy (CE), collision cell exit potential (CXP), and entrance potential (EP) were first optimized by direct flow infusion analysis of the EA standard solution. The results indicated that the negative mode was more favorable than the positive ion mode (better signal to noise ratio in the real sample). In negative full scan spectra, one precursor ion (*m*/*z* 301) and several product ions including *m*/*z* 257 (loss of CO_2_), *m*/*z* 229 (loss of CO_2_ and CO), and *m*/*z* 185 (loss of two CO_2_ and one CO) were observed (Figure 1). The transition *m*/*z* 301/257, 301/229, and 301/185 were selected to analyze the standard solution in multiple reaction monitoring (MRM) mode, which revealed that transition *m*/*z* 301/229 showed the highest intensity. Therefore, the *m*/*z* 301/229 transition was selected for MRM-based quantification.

To obtain a short run time and adequate resolution, LC conditions such as mobile phase, column, injection volume, and flow rate, which could significantly influence the separation and retention times, were optimized. This revealed that a short ZORBAX SB-C18 column (4.6 mm × 50 mm, 1.8 μm) could significantly decrease the run time, and that acetonitrile rather than methanol decreased the background noise and resulted in the best resolution. More importantly, when formic acid was added into the mobile phase as a modifier, the sensitivities, retention behaviors, and peak shape of EA were markedly improved. Hence, 20% acetonitrile in 0.1% formic acid aqueous solution was selected as the mobile phase.

### 2.3. Method Validation

#### 2.3.1. Selectivity and Matrix Effect

**Figure 2:**
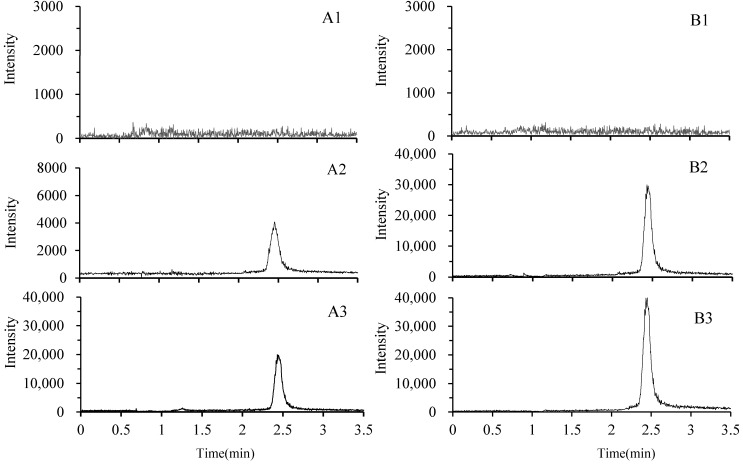
Typical UPLC-MS/MS chromatograms of ellagic acid in rat plasma (**A**) and liver (**B**). (**A1**) blank plasma; (**A2**) blank plasma spiked with ellagic acid (10 ng/mL); (**A3**) plasma 0.5 h after oral administration of a single dosage 50 mg/kg ellagic acid; (**B1**) blank liver sample; (**B2**) blank liver sample spiked with ellagic acid (100 ng/g); (**B3**) liver sample 0.5 h after oral administration of a single dosage of 50 mg/kg ellagic acid.

To assess the endogenous interference for sample analysis, the chromatograms derived from blank samples, samples spiked with EA, and samples after EA consumption were inspected. The typical chromatograms of blank plasma and liver samples, plasma and liver samples spiked with EA, and plasma and liver samples after EA consumption are shown in Figure 2. Under the UPLC-MS/MS conditions described above, the retention time of EA was about 2.4 min, and no interference peak was detected at related retention times in blank plasma, tissues, and samples after EA consumption. The matrix effect values for EA at different levels were 97.3%–104.3%, with RSD 3.1%–5.6%, respectively, indicating that no significant matrix effect was observed in plasma and tissue samples under the present experimental conditions. Under these optimized conditions, EA was eluted at about 2.4 min, and the total run time was less than 3.5 min. Therefore, it could be applied to high-throughput assays.

#### 2.3.2. Linearity and the Lower Limits of Quantitation

A linear relationship was found between the peak area and EA concentrations within the concentration ranges of 10–1,280 ng/mL. The typical equation of the calibration curves was *Y* = 523.51*X* – 2,478.2, where *Y* represented the EA concentration (ng/mL) and X represented the EA peak area. The coefficient of determination (*R*^2^) was found to be 0.9986. The lower limits of quantitation (LLOQ) were 5.0 ng/mL or 2.5 ng/g for EA in plasma or tissues, respectively.

#### 2.3.3. Precision and Accuracy

The precision and accuracy were assessed by determining quality control (QC) samples (*n* = 5) at three levels of concentrations, and the results were provided in Table 1. The accuracy values ranged from −5.3% to 4.6% and the intra- and inter-day precisions were 3.0% to 5.7% and 3.6% to 7.2%, respectively. These results indicated that the present UPLC-MS/MS method was accurate, reliable, and reproducible.

**Table 1:** Accuracy, precision, recovery, and stability of ellagic acid in rat plasma and liver samples.

Sample	Level ng/mL	Accuracy	Precision (RSD %)		Recovery %	Stability %		
		RE %,	Intraday	Inter-Day		Short Term *^a^*	Long Term *^b^*	Freeze–Thaw *^c^*
		*n* = 5	*n* = 5	*n* = 15				
plasma	50	−5.3	5.7	7.2	103.3 ± 6.4	93.2 ± 3.6	92.6 ± 6.4	90.5 ± 7.8
	200	3.2	4.1	3.6	95.3 ± 3.2	94.7 ± 5.2	93.6 ± 6.2	92.6 ± 8.0
	800	−2.8	3.0	4.0	98.2 ± 4.6	94.9 ± 4.5	94.4 ± 5.7	93.2 ± 6.9
liver	50	−3.5	4.5	6.8	90.4 ± 8.7	92.6 ± 3.6	89.6 ± 8.7	90.4 ± 7.4
	200	−1.8	4.1	4.3	94.5 ± 5.2	93.7 ± 6.5	93.3 ± 6.3	91.3 ± 6.9
	800	4.6	3.2	4.7	96.8 ± 4.7	95.1 ± 5.0	91.5 ± 5.7	92.4 ± 3.6

*^a^* Short-term stability of EA was estimated at room temperature for 24 h; *^b^* Long-term stability was studied following 2 weeks of storage at −20 °C; *^c^* Freeze–thaw stability was evaluated at three consecutive freeze–thaw cycles (−20 °C to room temperature).

#### 2.3.4. Recovery and Stability

The recoveries of EA at three QC concentration levels (*n* = 5) in plasma and tissues were 90.4%–103.3% (Table 1). The samples were found to be stable after being placed at room temperature for 24 h, stored at −20 °C for 2 weeks, or subjected to freeze–thaw cycles in rat plasma and tissues. In addition, the treated samples were stable at 4 °C in an autosampler for 12 h, and the results of stability were found to be within the range of 90.7% and 102.1% for EA in plasma and tissues, indicating that a large number of samples could be determined in each analytical run. Based on above results, a reliable, reproducible, and robust method has been developed and validated.

### 2.4. Pharmacokinetics and Tissue Distribution

**Figure 3:**
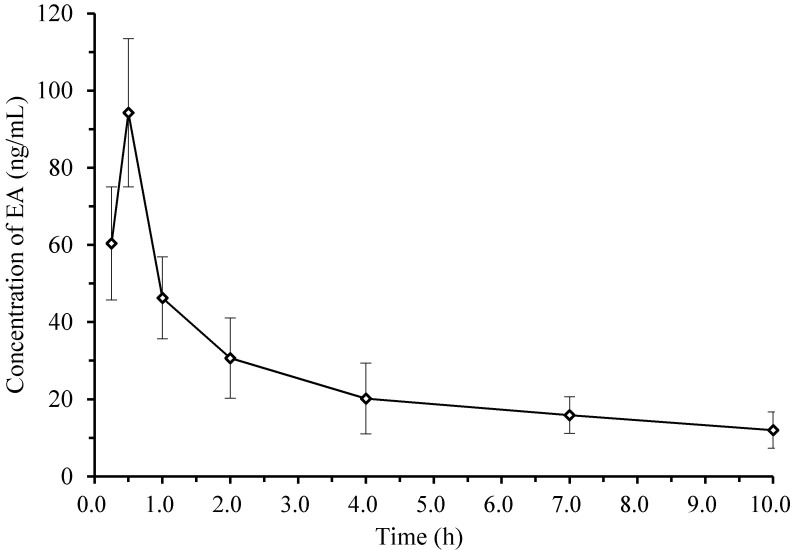
Mean plasma concentration–time profiles of ellagic acid in the Sprague–Dawley rats after consumption of ellagic acid at doses of 50 mg/kg (Means ± SD, *n* = 5).

The developed method was successfully employed to determine EA concentrations in rat plasma and tissue samples after consumption of EA at a single dose (50 mg/kg). The mean plasma concentration-time curves of EA in plasma are shown in Figure 3. The compartment model was established using the survival square sum (SUM), Akaike’s information criterion (AIC), and the fitted degree (*γ*^2^). A two-compartment mode (weight = 1) was the best fit for the plasma concentration-time curves, and the main pharmacokinetic parameters from two-compartment analysis were listed in Table 2. After the oral administration of EA dose at 50 mg/kg, the maximum concentration (*C*_max_) of EA in plasma was 93.6 ng/mL (0.31 μM), and the area under curve (*AUC*_0–∞_) of the concentration-time profile was 457.2 ng/mL × h, indicating that the absorption of EA was extremely poor. The values of *t*_max_, *t*_1/2α_, and *t*_1/2β_ were 0.5 h, 0.25 h, and 6.86 h, respectively, suggesting that EA entered the central compartment (circulation) in a rapid pattern, then distributed from the central compartment quickly into the peripheral compartments, and finally was eliminated or metabolized in a moderately slow pattern. The pharmacokinetic profiles of EA obtained in the present study were consistent with those reported previously [7,14].

**Table 2:** The main pharmacokinetic parameters after oral administration of ellagic acid at doses of 50 mg/kg in rats.

Parameters	Unit	Mean ± SD
*t*_1/2_α	h	0.25 ± 0.02
*t*_1/2_β	h	6.86 ± 0.05
CL/F	L/h/kg	109.3 ± 11.7
*AUC*_0–t_	ng/mL × h	252.0 ± 17.0
*AUC*_0–∞_	ng/mL × h	457.2 ± 34.0
K10	1/h	0.54 ± 0.04
K12	1/h	1.90 ± 0.19
K21	1/h	0.47 ± 0.05
Ka	1/h	14.52 ± 1.87
*C*_max_	ng/mL	93.6 ± 31.0

**Figure 4:**
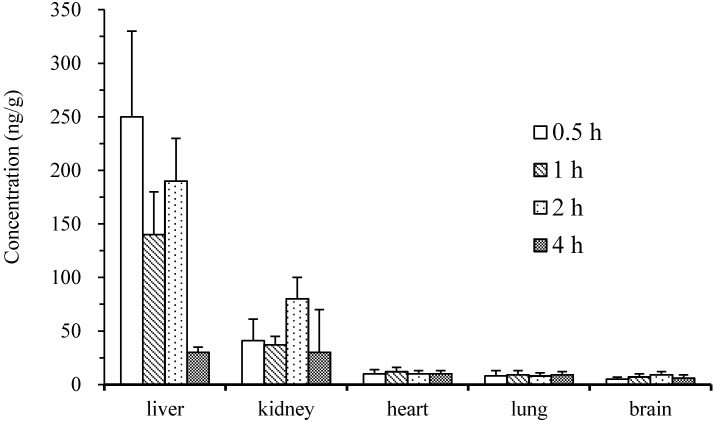
Distribution of ellagic acid in liver, kidney, lung, heart, and brain after 0.5, 1, 2, and 4 h following oral administration of EA at a dose of 50 mg/kg (*n* = 5).

Tissue distribution in rats at 0.5, 1, 2, and 4 h after EA consumption are presented in Figure 4. These data show that EA is distributed widely in many tissues including heart, liver, kidney, and even brain. However, the concentration was relatively low except for in the liver and kidney. At 0.5 h after EA consumption, the EA concentration in liver peaked, and the concentration was three-fold higher than the maximum concentration in plasma. Kidney contained moderate amounts of EA and tissue extracts from the heart, lung, and brain contained low concentration. At 4 h after oral administration, tissue extracts from the liver and kidney still had comparatively high concentrations of EA, whereas extracts from heart, lung, and brain had relatively low concentrations of EA. Notably, EA was detected in the brain at a relatively low concentration, suggesting that EA could cross the blood–brain barrier. However, the EA distribution results in the lung were contradictory with those described previously, which reported that EA accumulated preferably in the lung [5]. 

## 3. Experimental Section

### 3.1. Chemicals and Reagents

The analytical standard of EA was purchased from the National Institute for the Control of Pharmaceutical and Biological Products (Beijing, China), and EA samples for oral consumption of analytical grade were purchased from Sigma-Aldrich Co. (St. Louis, MO, USA). HPLC-grade acetonitrile, formic acid, and methanol were obtained from Merck (Darmstadt, Germany). Ultrapure HPLC-grade water was prepared using the Milli-Q^®^ system (Millipore, Milford, MA, USA). ProElut^TM^ PLS SPE cartridges (30 mg, 1 mL) were supplied by Dikma Technologies Inc. (Beijing, China).

### 3.2. Drug Administration and Sample Preparation

To develop a protocol for sample pre-preparation with high efficiency and improved recovery, different methods were compared. Female Sprague–Dawley (SD) rats (180 ± 20 g) were purchased from the Laboratory Animal Center of the Academy of Military Medical Sciences, Beijing, China. All rats were housed in an environmentally controlled room with temperature maintained at 22 ± 3 °C, 30%–60% relative humidity, and 12 h light/dark cycle. They were fed with standard laboratory food and water *ad libitum*. Prior to the experiments, animals were quarantined and allowed to acclimate for 1 week. Throughout the experiments, animals were monitored and maintained in accordance with the Guide for the Care and Use of Laboratory Animals (National Research Council of the USA, 1996) and related ethical regulations of Beijing Forestry University. For pharmacokinetic and tissue distribution studies, 35 rats were evenly and randomly divided into seven groups and then orally administered EA (suspended in 0.5% carboxymethyl cellulose sodium solution at a concentration of 20 mg/mL) at a dose of 50 mg/kg. These animals were fasted for 12 h with free access to water before the experiment. At 0.25, 0.5, 1, 2, 4, 7, and 10 h after EA consumption, respectively, one group of rats was killed under ether anesthesia, the blood was collected from the fossa orbitalis vein, and centrifuged (TGL-16G; Shanghai Precision Instruments Co., Ltd., Shanghai, China) at 2500 rpm for 15 min at 4 °C, and the supernatants were collected. The brains, hearts, livers, spleen, lungs, and kidneys were collected and stored at −80 °C. Afterward, 15 μL of 50% phosphate (H_3_PO_4_) solution and 175 μL of 1 M potassium dihydrogen phosphate (KH_2_PO_4_) were added to 0.5 mL of plasma, successively. After vortex mixed for 1 min, the above mixture was separated by centrifugation at 8000 rpm for 5 min, and the supernatant was collected and stored at −80 °C for SPE extraction. Tissue samples were flushed with normal saline, blotted dried, and weighed, then 1.0 g of tissues was homogenized in 6 mL of normal saline. Next, 180 μL of 50% H_3_PO_4_ solution and 2.0 mL of 1 M KH_2_PO_4_ aqueous solution were successively added to the homogenate. Subsequently, the homogenate mixture was centrifuged at 8000 rpm for 5 min, and the supernatant was collected and stored at –80 °C for further SPE extraction.

### 3.3. Solid Phase Extraction

Prior to sample loading, all ProElut^TM^ PLS SPE cartridges were preconditioned with 3 mL of methanol and 3 mL of deionized water successively. On the day of analysis, preprepared plasma or tissue samples were thawed at room temperature, after which all samples were loaded onto preconditioned SPE cartridges. The loaded cartridges were then washed with 3 mL of deionized water and 3 mL of washing solution (0.1% formic acid aqueous solution: methanol 60:40, v/v). Finally, the prewashed cartridge was eluted with 0.5 mL elution solution (0.1% formic acid aqueous solution: methanol 10:90, v/v), after which the elution was collected and filtered through a 0.22-μm disposable syringe filter and injected into the UPLC-MS/MS system for analysis. These procedures were performed with a 24-port vacuum SPE manifold (Supelco, Bellefonte, PA, USA).

### 3.4. Instrumentation for LC and MS

An HPLC-DAD system (LC-20AT; Shimadzu, Tokyo, Japan) equipped with an autosampler (SIL-20A), a DAD detector (SPD-M20A), a communications bus module (CBM-20A), two liquid chromatographs (LC-20AT), and a column oven (CTO-10AS) were used to optimize the pre-preparation of blood or tissues with SPE. Samples were analyzed on a Diamonsil RP-C18 column (250 mm × 4.6 mm I.D., 5 μm; Dikma Technologies Inc., Lake Forest, CA, USA) at 30 °C, with a detection wavelength of 254 nm. The mobile phase was a mixture of 0.2% phosphate/water (80%) and acetonitrile (20%) with a flow rate of 1.0 mL/min.

EA in rat plasma and tissues was quantified on an Agilent 1260 UHPLCUPLC system (Agilent Corp., Santa Clara, CA, USA) with an AB SciexQTrap 5500 tandem mass spectrometer (AB Sciex, Framingham, MA, USA). The UHPLCUPLC system (Agilent Corp.) was equipped with a quaternary pump, an online vacuum degasser, an autosampler, an automatic thermostatic column oven, and an Infinity series diode array detector (DAD; Agilent Corp.). Analytical separation was performed on an Agilent ZORBAX SB-C18 column (4.6 mm × 50 mm, 1.8 μm) at 35 °C with a flow rate of 0.6 mL/min. The mobile phase was a mixture of 0.1% formic acid/water (70%) and acetonitrile (30%). The injection volume of each sample was 5 μL, and prior to initial sample injection, the column was equilibrated with the mobile phase at the above flow rate for a minimum of 15 min. The AB SciexQTrap 5500 mass spectrometry was operated in negative electrospray ionization (ESI) mode using the following instrument settings: curtain gas (CUR), 30 mL/min; collision-activated dissociation (CAD), medium; IS, −4500.00 V; TEM, 600.00 °C; DP, −80.00 V; CE, −42 V; CXP, −19 V; EP, −10 V. These settings were optimized by direct infusion of 400 ng/mL of EA at 10 μL/min flow rate using the integrated syringe pump and then refined using the *Compound Optimization* feature of the Analyst software (AB Sciex) using flow injection analysis. EA was then quantified in MRM mode using the following mass transitions: *m*/*z* 301/229. Data were acquired and processed using AB Sciex Analyst software (version 1.6.1).

### 3.5. Method Validation

#### 3.5.1. Preparation of Standard Solutions, Plasma and Tissue Calibrators, and Quality Controls

Standard stock solution of EA was dissolved in methanol at concentration of 100 μg/mL. Working standards were prepared by dilution of the stock solution in methanol to obtain the desired concentrations of 100, 200, 400, 800, 1600, 3200, 6400, and 12,800 ng/mL for EA. Plasma or tissue calibrators were prepared individually by mixing 10 μL of working standard solution with 90 μL of blank plasma or blank tissue normal saline homogenate solution. Samples were then pre-prepared and extracted with the SPE cartridge, as described above. The QC samples containing 50, 200, and 800 ng/mL of EA were prepared in a manner similar to that used for preparing the calibrator samples.

#### 3.5.2. Specificity and Matrix Effect

Specificity was assessed by analyzing blank plasma and tissue homogenate samples, blank plasma and tissue homogenate samples spiked with EA standard, and rat plasma and tissue homogenate samples after EA consumption. All samples were prepared with ProElut^TM^ PLS SPE. The matrix effect was defined as the ion suppression/enhancement of the ionization of EA, which was evaluated by comparing the corresponding peak area of the deproteinized samples of blank plasma from six rats spiked with EA at 50, 200, and 800 ng/mL to those of neat standard solutions at equivalent concentrations; this peak area ratio is defined as the matrix effect.

#### 3.5.3. Linearity, Accuracy, and Precision

The calibration curves were constructed from the peak area of each standard solution against the nominal concentrations using eight-level nonzero standards and a linearly weighed (1/x) least squares regression model. The calibration curve required a correlation coefficient (*R*^2^) of 0.99 or better. Method accuracy was estimated by calculating the percent deviation observed in the analysis of QC samples and expressed by relative error. Intraday precision was estimated by analyzing QC samples of plasma or tissue homogenates at three concentrations within 24 h (*n* = 5). Inter-day precision was estimated by repeated analysis of QC samples over 3 consecutive days (*n* = 15). The variability in determination was expressed as the relative standard deviation (RSD, %) and the accuracy was expressed as the relative error (RE, %). The LLOQ was defined as the lowest concentration that could be determined with both RE and RSD within 20% [15].

#### 3.5.4. Recovery

The recoveries of EA from rat plasma and tissues were determined by comparing peak area ratios from regularly pretreated QC samples with those obtained from the direct injection of pure standard solutions at three QC concentration levels. The recoveries of EA in rat plasma or tissue homogenates were examined at least three times.

#### 3.5.5. Stability

The stabilities were examined by evaluating small variations under three different conditions. All stability studies were assayed at 50, 200, and 800 ng/mL levels. Short-term stability of EA in different QC samples at room temperature was estimated after 24 h. Long-term stability was studied by assaying samples following 2 weeks of storage at −20 °C. Freeze–thaw stability was evaluated at three consecutive freeze-thaw cycles (−20 °C to room temperature). The results were expressed as the percentage of initial content of EA in the freshly treated samples. Samples were considered stable if assay values were within the acceptable limits of accuracy (±15% RE) and precision (±15% RSD).

#### 3.5.6. Data Analysis

Data were collected and analyzed with Excel 2010 (Microsoft, Redmond, WA, USA) and SPSS V19.0 (SPSS Inc., Chicago, IL, USA). Pharmacokinetic parameters including *C*_max_, *T*_max_, and *AU*C_0–__∞_, and the compartment model were analyzed by the Drug and Statistics (DAS) 3.0 software (Chinese Mathematical Pharmacology Society, Beijing, China).

## 4. Conclusions

The present study developed a rapid, sensitive, reproducible and validated UPLC-MS/MS analytical method for EA determination in plasma and tissues of rats. Furthermore, a sample cleanup protocol with SPE technique was also developed prior to the UPLC-MS/MS analysis. To the best of our knowledge, this is the first study reporting the use of SPE for EA preparation from plasma and tissues in pharmacokinetic studies. Compared to conventional sample preparation techniques, SPE has many advantages such as easy handling, high-throughput, time-saving, and high-recovery properties. The SPE sample cleanup protocol and the UPLC-MS/MS analytical method were then successfully applied to the pharmacokinetic and tissue distribution studies of EA. It revealed that the absorption of EA was extremely poor after oral administration, and its pharmacokinetic profile fitted to a two-compartment model. In all examined tissues including kidney, liver, heart, lung and brain, EA was detected, but much higher levels of EA were found in kidney and liver.
